# A Two-Step Guided Waves Based Damage Localization Technique Using Optical Fiber Sensors

**DOI:** 10.3390/s20205804

**Published:** 2020-10-14

**Authors:** Rohan Soman, Kaleeswaran Balasubramaniam, Ali Golestani, Michał Karpiński, Pawel Malinowski

**Affiliations:** 1 Institute of Fluid Flow Machinery, Polish Academy of Sciences, Fiszera 14, 80-231 Gdańsk, Poland; kaleeswaranb@imp.gda.pl (K.B.); pmalinowski@imp.gda.pl (P.M.); 2 Faculty of Physics, University of Warsaw, Pasteura 5, 02-093 Warszawa, Poland; Ali.Golestani@fuw.edu.pl (A.G.); mkarp@fuw.edu.pl (M.K.)

**Keywords:** guided waves, fiber Bragg grating (FBG) sensors, damage detection, damage localization, edge filtering

## Abstract

Structural health monitoring (SHM) systems help in reducing maintenance cost and avoiding catastrophic failure of the structure. As a result, they have been a focus of research for the past few decades. Ideally, the methods employed should be low cost and able to detect and localize small levels of damage reliably and accurately. This paper describes a guided waves (GW) based two-step technique for damage detection and localization using fiber Bragg grating (FBG) sensors. The FBG sensors offer benefits such as the ability to be embedded and multiplexed as well as being lightweight and insensitive to electric and magnetic fields, and they have long been seen as a promising solution for the GW measurements in structures. Unfortunately, in the conventional wavelength-based interrogation they have very low signal to noise ratio and as a result low sensitivity. Therefore, the FBG sensor is incorporated in the edge filtering configuration. The major challenges in the use of FBG sensors for GW-based detection are their directional sensitivity and passive nature. The passive nature leads to the reduction in the available actuator–sensor (AS) pairs while the directionality makes the signal processing a challenge. The proposed two-step methodology overcomes these shortcomings of FBG sensors. In the first step the amplitude weighted elliptical approach is used to identify the hotspots due to the inadequate number of AS pairs, the elliptical approach is not sufficient for damage localization. Therefore, in order to further localize the damage the edge reflection based ray-tracing approach is implemented in the second step. Through the two step method, the damage is accurately located. The paper provides the proof of concept of the proposed methodology on an aluminum plate with simulated damage. The results indicate, that indeed the two-step methodology allows accurate damage localization and overcomes the possibility of false detections.

## 1. Introduction

All structures have a limited lifespan and are prone to structural defects like corrosion, fatigue, wear, delamination, etc. The aim of structural health monitoring (SHM) techniques is to detect the defects by analyzing the in-service performance of these structures. Typically, a SHM system consists of sensors for data collection, some signal preprocessing for improving the quality of the data collected, and signal processing for extracting the damage sensitive feature in order to make a decision on the condition of the structure. Several techniques, such as vibration-based techniques [1], strain-based techniques [2], guided waves (GW)-based techniques [3], electromechanical impedance-based techniques [4], etc., have been proposed in literature, making use of different types of sensors as well as signal processing techniques to reliably detect and locate damage. These methods have been applied successfully for experimental validation but for real applications they have not satisfied all the service requirements. As a result the search for a low-cost technique is still ongoing.

GW-based techniques have been popularly employed for plate-like structures, as they offer the possibility to reliably detect and localize the damage. Traditionally piezoelectric materials such as lead zirconate titanate (PZT) are used for the actuation of the GW in structures. In addition to the PZT, other piezoelectric materials, macro-fiber composites, magnetorestrictive transducers, electromagnetic acoustic transducers, etc. have been used in the literature as well [5]. For the sensing, in addition to the above mentioned transducers the scanning laser Doppler vibrometer (LDV) and optical fiber sensors such as Fabry–Pérot interferometers and fiber Bragg grating (FBG) sensors have been used in the literature [6,7]. The LDV offers possibility for full wave field measurements and hence gives a large amount of information, but unfortunately, measurements with LDV are time consuming and as a result not always possible in in-service conditions. The other sensing techniques such as PZT transducers have been employed extensively due to their low cost but have issues related to sensitivity to electric and magnetic fields. The optical fiber sensors are immune to the magnetic and electric fields. Moreover, due to their small size, ability to be embedded in the structure, ability to be multiplexed have been looked at as an ideal solution for the GW sensing. Unfortunately, in their conventional configuration based on change in the wavelength, their sensitivity to GW is very low. In order to overcome this low sensitivity a novel approach of employing the FBG sensor in the edge filtering configuration has been proposed [8]. The edge filtering approach makes use of amplitude based detection and allows signal amplification which improves the sensitivity of FBG sensors to GW. The edge filtering approach has been explained in detail in [8,9].

Although, the FBG sensors in the edge-filtering approach offer the potential to measure GW, their use for damage detection and localization is not a simple extension of the signal processing methods for the PZT based sensors. The reason for this is two-fold: (a) the FBG sensors are passive sensors and (b) the FBG sensors have directional sensitivity to the incident GW. Due to the passive nature of the FBG sensors, the tomography-based techniques or the phased array-based techniques such as the ones proposed in [10,11,12,13] cannot be directly applied to the FBG sensors, as the number of sensor–actuator pairs available is severely reduced. For instance, for a 6-sensor system consisting of six PZT sensors there are potentially 62 = 15 sensor–actuator pairs, at the same time, for a sensor system with five actuators and one FBG sensor there are only five sensor–actuator pairs. Moreover, it has been shown in [14,15,16] that the response of FBG’s to incident GW is a function of cosine-squared of the angle between the axis of the FBG and the incident wave. Thus, the signal processing technique should incorporate these FBG imposed shortcomings into the damage detection algorithm.

Despite these systematic shortcomings of the FBG sensors, due to the other advantages offered by the FBG, the authors strongly believe that FBG-based GW sensing is a very promising tool for GW-based damage detection. The paper presents a two-step methodology for damage detection and localization. In the first step, the hotspots of damage are detected using an ellipse based approach. These identified hotspots are then investigated with the edge reflection approach in order to determine the damage location accurately. It is noted that, with 3 or more actuator-sensor (AS) pairs, the elliptical method in itself will be enough for damage localization. But in the case of FBG sensors, as they are passive, the number of AS pairs is greatly reduced. In such case there might be areas on the structure where it is not possible to localize the damage using elliptical method alone. This is the primary motivation for the development of the two-step methodology. The innovation of the paper is the development of a methodology which may be applied to structures or parts of structures where three AS pairs are not present for the triangulation of the damage location. This ensures a wider applicability of the method. Also, the proposed method overcomes the above mentioned constraints imposed by the use of FBG sensors. The paper presents validation on an aluminum plate. The results indicate that indeed the two-step method can overcome the specific challenges associated with the use of FBG for GW based damage detection.

The rest of the paper is organized as follows. The next section describes the two-step methodology for damage detection. Section 3 presents the specimen used for the validation. Section 4 presents and discusses some results. Finally, in Section 5, conclusions are drawn based on the presented results and the areas of future work have been identified.

## 2. Methodology and Implementation

### 2.1. Hotspot Identification

**Figure 1:**
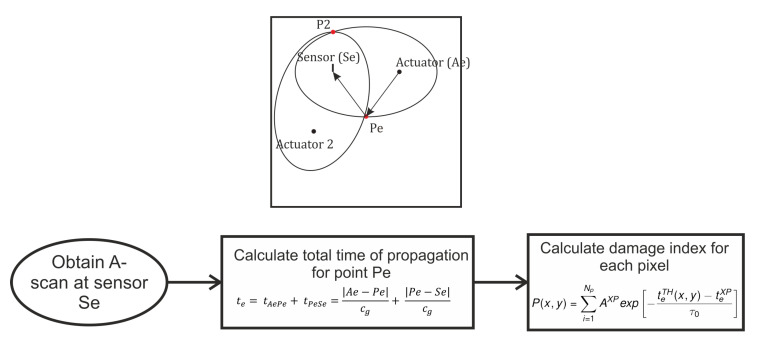
Amplitude weighted ellipse approach for damage hotspot identification.

The most common technique for damage localization is through triangulation of differential signals using three actuator–sensor pairs. Several damage visualization techniques have been proposed in the literature [5]. In this work we employ an amplitude weighted elliptical approach, which is a modification of the work carried out in [17]. The amplitude weighing is required to incorporate the directionality of the FBG sensors. Figure 1 shows the concept concisely.

The structure is divided into pixels. For each pixel on the structure, the theoretical time of flight (TOF) is calculated based on the group velocity $c_{g}$ and the summation of distance of the path-actuator-investigated point (|Ae−Pe|) and investigated point-sensor (|Pe−Se|), where || denotes euclidean distance. For the experimental data, the differential data (difference in baseline and investigated state) is used, a Hilbert envelope is fitted to the difference, and the peaks identified. Each peak corresponds to the maxima of a wave packet. The correlation for each of the peaks with the theoretical TOF is then calculated. If the theoretical TOF agrees with the value of the peak, a high value is obtained at that pixel. This value is further weighted by the amplitude of chosen peak. The final damage index for each pixel is the summation of all values for all the AS pairs for the same pixel as shown in equation.

(1) $$P\left(x,y\right)=\sum_{i=1}^{N_{p}}Amp^{XP}exp\left[-\frac{t_{e}^{TH}\left(x,y\right)-t_{e}^{XP}}{\tau_{0}}\right]$$

where P(x,y) denotes pixel value (Damage indicator), $N_{p}$ denotes number of AS paths, Amp denotes amplitude, *t* denotes time of arrival, and $\tau_{0}$ is a decay factor playing the role of a decay rate of an exponential windowed function applied to reduce secondary reflections of the scattered signal which is taken as 5 μs similar to [17]. The superscripts XP and TH refer to experimental measurements and theoretical estimation, respectively.

As can be seen, when there are 2 AS pairs in Figure 1, the intersection point of the ellipses are not unique, as a result, the damage index at Pe and P2 will show similar values. As a result the elliptical approach with only 2 AS pairs allows us to identify the possible locations of damage. In order to further localize the edge reflection based approach using ray tracing needs to be used.

### 2.2. Damage Localization

**Figure 2:**
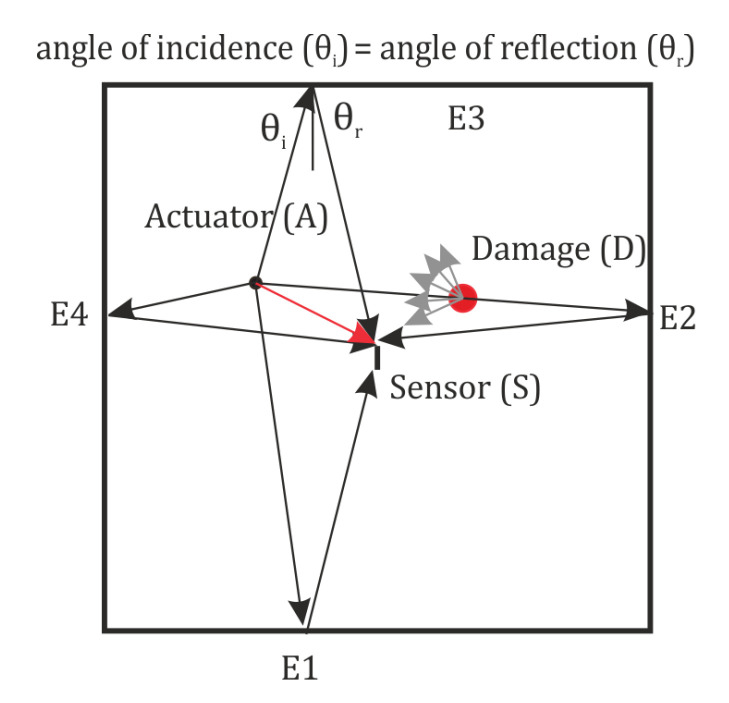
Concept of edge reflection-based ray tracing.

The FBG sensors are passive sensors, as a result, the number of AS pairs is reduced considerably. In order to maximize the area covered by the sensor network the edge reflection-based approach proposed in [18] is implemented. In the frequency range used in this study, only the zero order symmetric (S0) and antisymmetric (A0) modes are excited. The A0 mode has a much smaller wavelength and as a result is sensitive to small levels of damage and hence is used in this study [19]. For an A0 wave incident on the plate boundaries, no mode conversion occurs and the wave reflection has an angle equal to the angle of incidence. Thus, the propagating wave may be treated as a ray of light from the actuator to the edges, and from the edges to the sensor. If the ray of light encounters a discontinuity in the path, some of the energy will be scattered, as a result the amplitude of the light received at the sensor will be reduced. By identifying the paths which undergo the change in the amplitude the ray which encounters the damage in its path may be identified, which in turn will allow us to localize the damage accurately. Figure 2 shows the direct path between the actuator and sensor. It also shows the first order reflection from each of the 4 edges. The order refers to the number of edges the path is reflected from before reaching the sensor. The edge reflection paths which pass through the identified hotspot are identified. Knowing the group velocity of the excited wave and the total length of these reflected paths, the TOA of the wave can be estimated. The amplitude of the wave in the short prespecified window around the theoretical TOF may be used to determine if the damage location lies on the particular path. For instance, in Figure 2, the reflection paths A-S, A-E1-S, A-E3-S, and A-E4-S are unaffected by the damage, so the amplitude of the wave in the time window for the healthy and damage scenarios should ideally be same (subject to measurement errors and secondary wave reflections from the damage). On the other hand the path A-E2-S passes through the damage location, as result will be affected by the damage. Some of the wave energy is scattered by the damage, as a result, the amplitude of the wave at the sensor will be lower. This may be used to identify the exact location of the damage from the identified hotspots.

The effect of the damage on the wave following a path can be quantified by taking the ratio of the RMS of the measured signal for the ith path in the specified window with the RMS of the baseline (healthy) signal for the ith path in the same time window as shown in Equation (2).

(2) $$A_{i}=\frac{RMS_{i}^{damage}}{RMS_{i}^{healthy}}$$

If this value is lower than a certain threshold it can be concluded that the ith path passes through the damage location. This information of the circumferential location combined with radial location allows accurate damage localization. In this study, the threshold value is determined based on engineering judgment, but may be determined based on sensitivity study which is identified as an area of future work.

**Figure 3:**
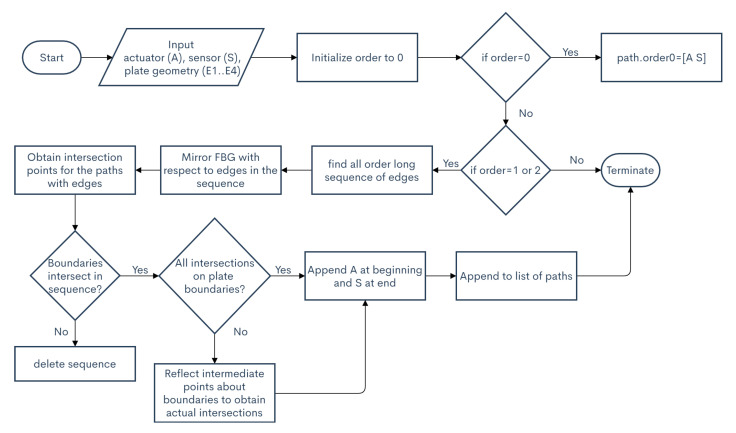
Flowchart for obtaining reflection paths.

**Figure 4:**
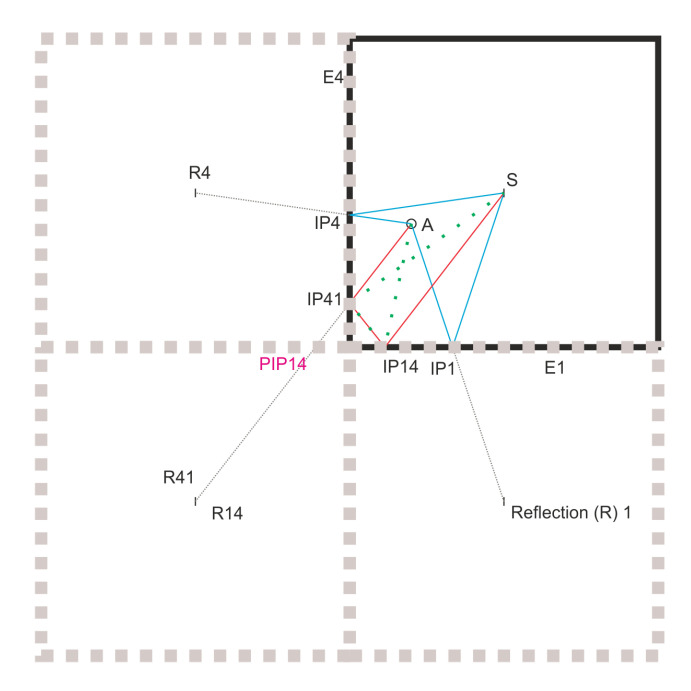
Example of order1 and order2 reflection paths.

The reflected paths are determined using the edges of the plate as mirrors and tracking the reflections about the different edges in a specified order. The flowchart for determining the paths of the edge reflections may be found in Figure 3 and can be explained using Figure 4.

As can be seen in Figure 4, the sensor (S) is reflected about E1 and E4 to obtain R1 and R4, respectively. These are the first order reflections. The first order path can be determined by connecting the actuator A and R1 and R4. The point of intersection of this line on E1 (IP1) and E4 (IP4), respectively, gives the intersection point which is used to define the order1 paths (blue). In order to obtain the order2 reflections, the reflections R1, and R4 are reflected about E4 and E1, respectively. In the case shown (as with the experiments presented later), the R14 and R41 are coincident due to the symmetric placement of S about the two edges. The line connecting the R41 and A allows us to get IP41. The second point of intersection (PIP14), is not on the true edge of the plate, in order to obtain the real point, it has to be reflected about E4. This gives us the point IP14, which completes the second order path A-IP41-IP14-S (red) for the reflection about the edges E4 and E1 respectively. As can be seen The path A-IP14-IP41-S (green) violates the law of reflection and is deleted from the path list.

### 2.3. Methodology Overview

**Figure 5:**
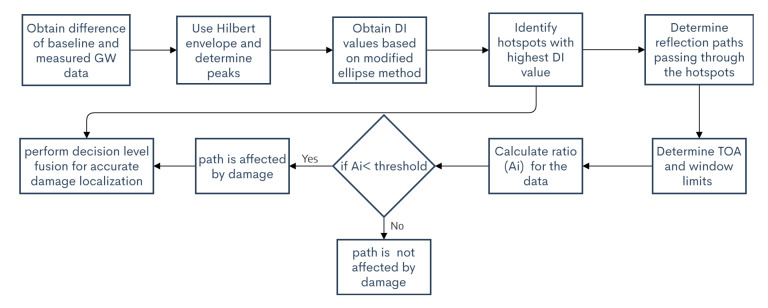
Flowchart of two step methodology.

The two step methodology uses information fusion at decision level. It is necessary to use the two steps to overcome false detection and localization which may be caused due to the lack of data. The flowchart for the two step methodology is given in Figure 5.

In the first step the hotspots are identified with the ellipse based approach, once the hotspots are identified, the reflection paths passing through the hotspots are determined, the theoretical TOA for the path is calculated, for obtaining the windows for determining the ratio $A_{i}$. If the value of $A_{i}$ is below the threshold, the hotspot through which the path passes is indeed the location of damage.

## 3. Experimental Setup

**Figure 6:**
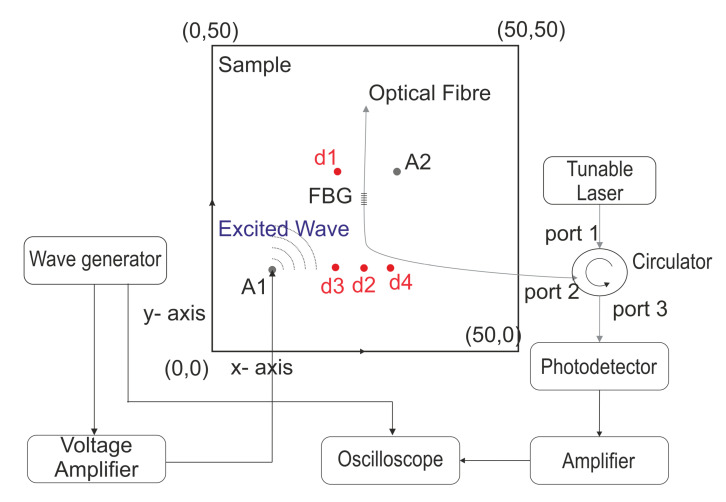
Schematic of the experimental set-up.

**Figure 7:**
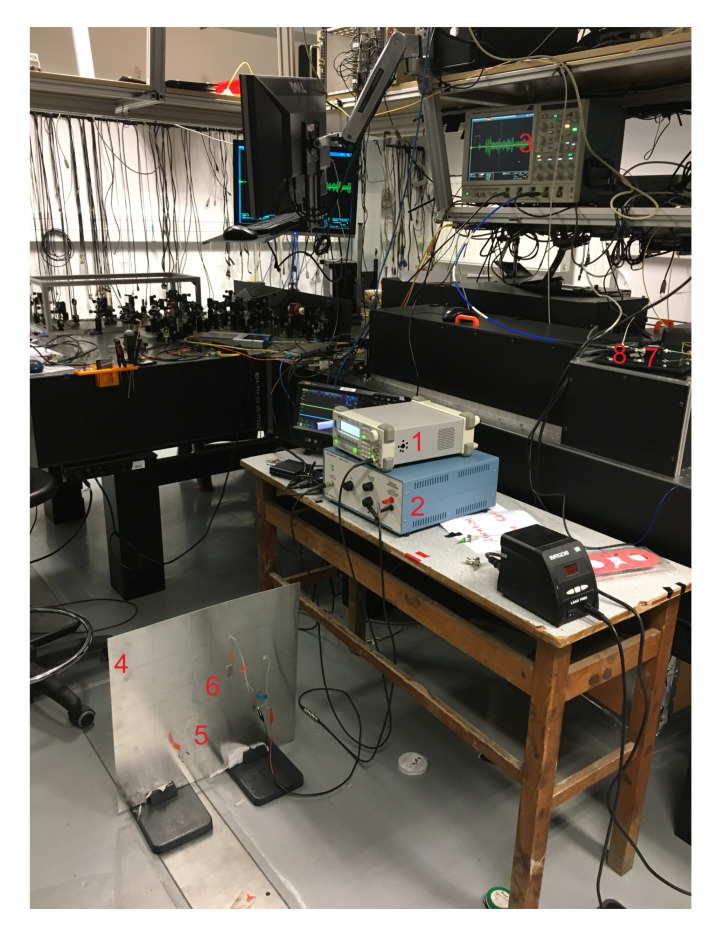
Experimental set-up. 1—wave generator; 2—voltage amplifier; 3—Oscilloscope; 4—plate; 5—PZT; 6—FBG; 7—photodetector; 8—amplifier.

The research presented uses FBG sensors in the edge filtering configuration for the damage detection. The excitation of the GW is carried out using PZT actuators. The schematic of the set-up is provided in Figure 6 and the actual set-up is shown in Figure 7.

A tunable distributed feedback, single-mode, fiber-coupled diode laser (Qphotonics, QDFBLD-1550-10, Ann Arbor, MI, USA) was used for generating a wavelength centered on the positive slope of the reflectivity of the FBG. The circulator channels the input from port 1 to port 2 and the reflected signal from the FBG (Femto Fibertech, 1556 nm, Goslar, Germany) to port 3. The reflected signal is detected by a biased photodetector (Thorlabs, DET01CFC, Bergkirchen, Germany), the response is amplified (1 kHz–1 GHz RF amplifier, 40 dB gain) and then collected through the oscilloscope (Tektronix, DPO7254C, Beaverton, OR, USA). Single mode SMF-28 fibers were used for all optical connections. On the mechanical part of the set-up, the arbitrary waveform generator (AWG, Rigol, DG1022, Beijing, China) was used to generate a 5-cycle Hann windowed sine at 50 kHz frequency. The voltage was amplified (20×) and applied to the PZT (CTS corporation, NCE51, Lisle, US). The generated GW were captured by the FBG. Synchronization between the mechanical, optical components and oscilloscope was achieved through triggering using the AWG. Since the signal was repetitive, averaging was used to improve the signal-to-noise ratio.

The sample of interest was an aluminum plate (50 cm × 50 cm × 0.1 cm). The FBG was attached using cyanoacrylate-based adhesive at the location (25,25). Two PZT actuators were attached using the same adhesive at locations A1 (10,20) and A2 (30,30). Four damage scenarios were simulated by using 2 neodymium magnets as added mass (ϕ = 2 cm, mass = 5.9 g each) at locations shown in the figure [D1: (20,30); D2: (25,20); D3:(20,20); D4: (30,20)]. The magnets were attached on either side of the plate. As the plate was thin and the magnets strong, a very firm attachment of the masses was possible. Each measurement was averaged 50 times in order to ensure robustness of the results.

## 4. Experimental Results and Discussion

### 4.1. Determination of the Group Velocity

**Figure 8:**
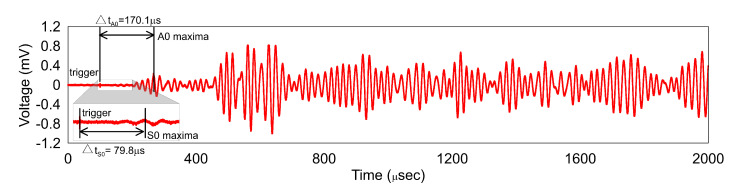
Time of arrival (TOA) for 50 kHz $S_{0}$ and $A_{0}$ waves excited at A1.

As explained in the methodology, knowing $c_{g}$ of the waves is imperative for accurate damage localization and hence was the first step. The excitation circuit (wave generator–voltage amplifier–PZT) has a certain delay at the time of the wave generation and the excitation of the PZT. This delay depends on the length of the cables and internal circuits of the voltage amplifier. This delay can be easily overcome by using a synchronized triggering for the measurement. For the optical circuit, the delay due to the optical fibers is negligible as both the measurements occur instantaneously. Figure 8 shows the time delay between the trigger and the maxima of the S0 and A0 wave, respectively. Knowing the frequency of excitation (50 kHz) and the number of cycles, the TOA can be calculated as 29.8 μs and 120.1 μs for S0 and A0 wave, respectively. For the distance between A1 and FBG, the velocity comes to 5310 m/s for S0 and 1320 m/s for A0 which are within 1% of those found from the dispersion curves [20].

A key point to note is that the S0 wave is weakly measured by the FBG sensor. The authors believe that the poor measurement performance of the FBG for the S0 wave is caused due to the relatively larger wavelength of the S0 wave and relatively small gauge length of the FBG sensor. This low sensitivity to $S_{0}$ wave has also been reported in [15]. The authors acknowledge that more investigation in this area is necessary using different frequencies for excitation as well as bonding lengths. The weak measurement of the S0 wave augurs well for our damage localization, as the A0 wave due to its smaller wavelength has the potential to detect small defects in the structure. Furthermore, as the S0 wave magnitude is low it can be neglected during the signal processing which simplifies the signal processing.

### 4.2. Hotspot Identification

**Figure 9:**
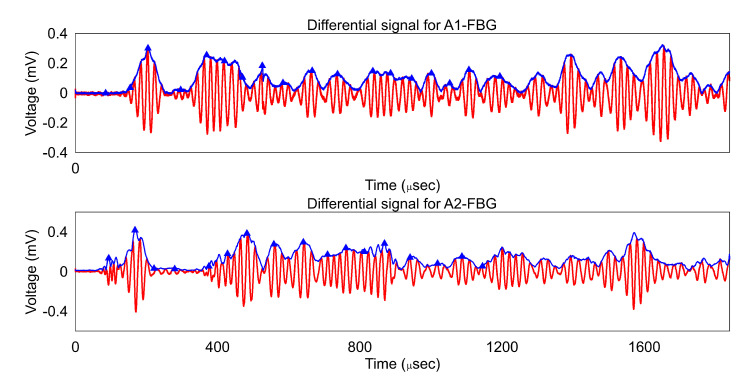
Differential signal with Hilbert envelope and identified peaks for scenario D2.

For the radial damage localization the plate was discretized in a grid of 500 × 500. The Equation (1) was then employed for determining the damage index (DI) for all the pixels using the $c_{g}$. For the estimation of the DI the experimental data was fitted with a Hilbert envelope and then peaks were identified. The identified peaks, the envelope, and the differential data (baseline signal− damaged signal) for damage scenario D2 for both AS pairs are shown in Figure 9.

It should be noted that, by taking the differential signal, the peaks due to the reflections from structural features such as edges are omitted. Furthermore, the time signal beyond 1200 μs was not considered as the measurements contain the higher order reflections as well as reflected fiber modes from the pigtails and ends which are not representative of the GW in the structure.

**Figure 10:**
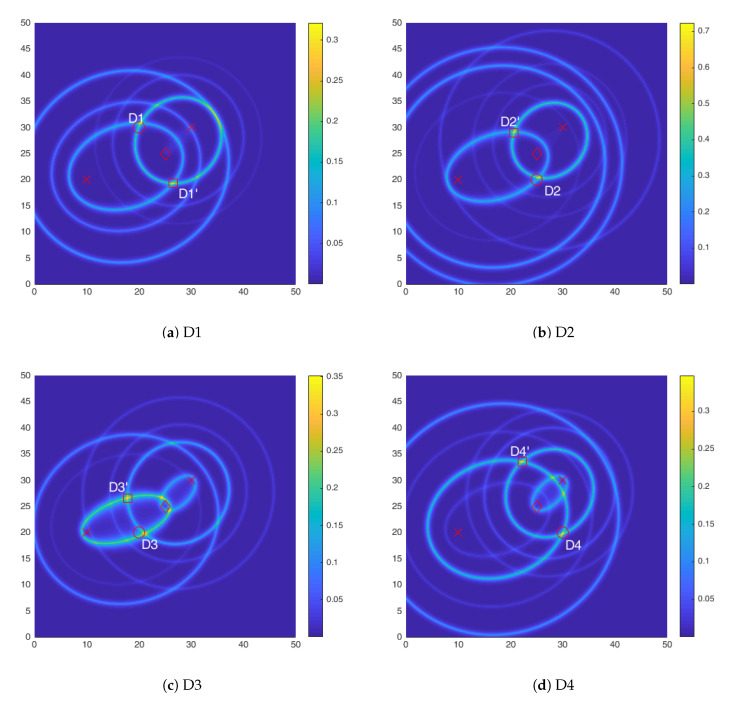
Hotspot identification using 2 AS pairs for all four damage scenarios. (**a**) D1; (**b**) D2; (**c**) D3; (**d**) D4.

Based on the identified peaks, the DI for each AS pair is calculated and fused in order to get a DI plot. Figure 10 shows the DI plots for all the four damage cases. As can be seen the damage is shown by a hotspot which agrees well with the location of damage. A point to note is that for the damage D2 the DI shows a much higher value than the other cases, this can be attributed to the directional nature of the FBG sensor, along the axial direction the sensitivity is highest and the reflected waves from the mass are in the axial direction thus accentuating the difference in the measurements.

It should be noted, that in the absence of the third actuator, there are at least two identified hotspots with high DI values. Therefore, the identified damage location is not unique and further damage localization to distinguish between the identified hotspots is necessary.

### 4.3. Damage Localization

**Figure 11:**
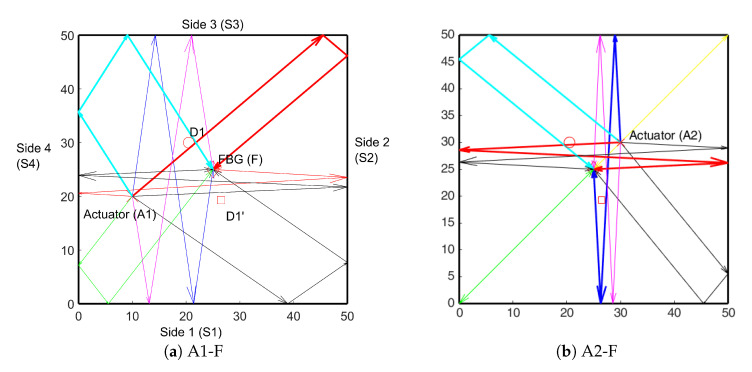
Nomenclature of structure and order 2 reflection paths for the AS pairs (**a**) A1 and (**b**) A2.

**Figure 12:**
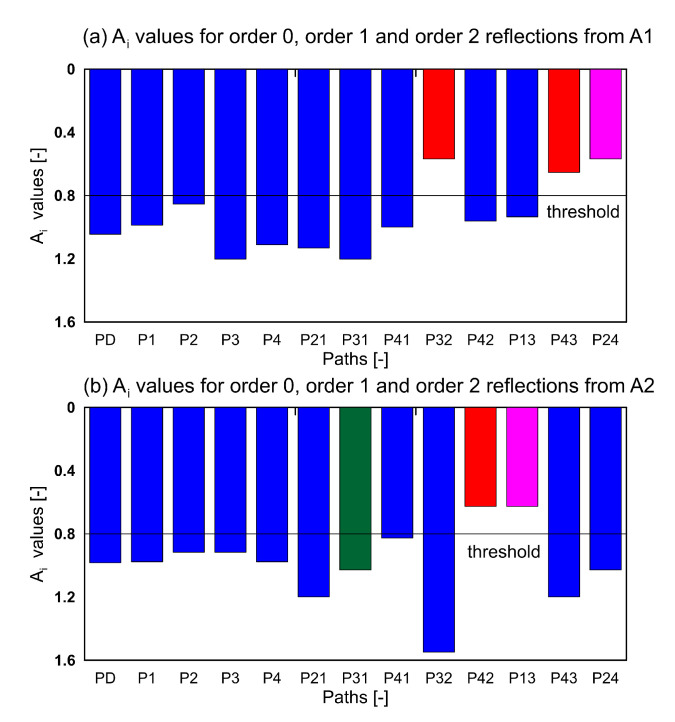
*A_i_* ratio for all identified edge reflection paths for both AS pairs.

The edge reflection approach was used to distinguish between the identified hotspots. In the first step for a given AS pair, all the possible edge reflection paths were identified. For the purpose of the study, only order0, order1, and order2 reflections were used. Figure 11 shows the order2 reflections (for clarity) for A1 and A2 and the FBG sensor. It can be seen that paths A1-S3-S2-F (red line in Figure 11a), A1-S4-S3-F (cyan line in Figure 11a) and A2-S4-S2-F (red line in Figure 11b) passes through one hotspot while path A2-S3-S1-F (blue line in Figure 11b) passes through the other hotspot. Equation (2) was used to obtain the ratio of the amplitudes for all the paths. The window for obtaining the RMS was determined as windowl = TOA + 5 μs and windowr = TOA + 55 μs. This ensures that the entire wave packet is included in the RMS calculations. The *A_i_* values for both actuators are shown in Figure 12.

The nomenclature of the paths and the bars is as follows. The bar P32 in Figure 12a corresponds to path A2-S3-S2-F. It can be seen that P32 shows the *A_i_* lower than the threshold (0.8), similarly the path A2-S4-S3-F (P43) has a value lesser than the threshold which shows that these paths are affected by damage and as can be seen they indeed pass through the location D1. The P24 (magenta) bar too shows a value lower than the threshold, but it does not pass through points D1 or D1’. The reason for the low value is that the TOA for P24 is exactly same as that for P32. This points to the need for the two-step methodology, as in the absence of the first step (hotspot location) the P24 will lead to a false damage localization. Similarly for the actuator2 (A2), the P42 and P13 show identical bars due to the same TOA. The P42 passes through point D1 and as a result is affected by the damage. On the other hand, the P13 does not pass through D1 or D1’. Again in absence of two-step detection the bar P13 would lead to faulty damage localization. The P31 (green), passes through the point D1’ and exceeds the threshold, so we can determine that the D1’ is indeed not the location of damage.

**Figure 13:**
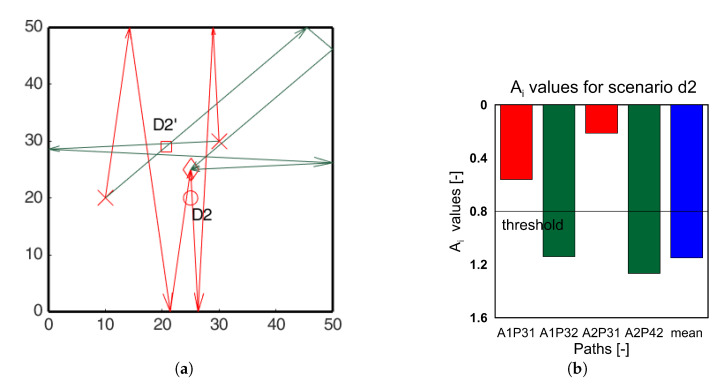
(**a**) Edge reflection paths passing through the identified hotspots; (**b**) *A_i_* ratio edge reflection paths passing through the identified hotspots for scenario D2.

**Figure 14:**
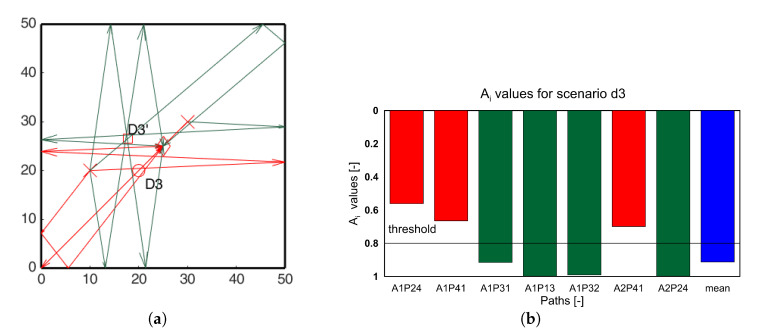
(**a**) Edge reflection paths passing through the identified hotspots; (**b**) *A_i_* ratio edge reflection paths passing through the identified hotspots for scenario D3.

**Figure 15:**
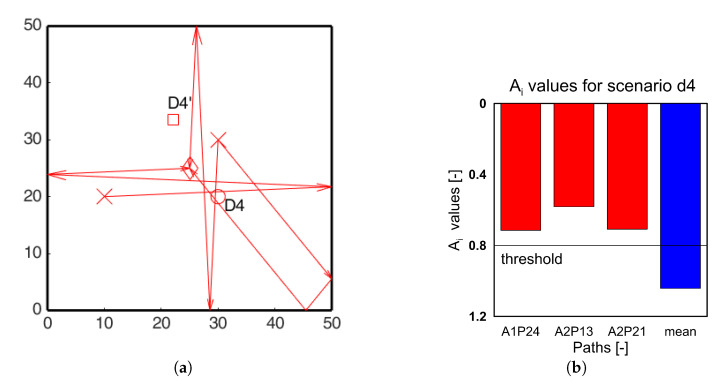
(**a**) Edge reflection paths passing through the identified hotspots; (**b**) *A_i_* ratio edge reflection paths passing through the identified hotspots for scenario D4.

Similar analysis was carried out for all the damage scenarios for both the actuators. For clarity only the paths passing through the identified hotspots are shown in the Figure 13, Figure 14 and Figure 15 for damage scenarios D2, D3, and D4, respectively. The bar charts show the *A_i_* values for the affected paths and the mean *A_i_* values of all paths (blue bars) for comparison. The threshold value for positive detection remains 0.8. In the bar chart, red bars correspond to paths passing through the damage, and green bars correspond to the other location. In all the cases, the red bars have values lower than the threshold, while the green bars exceed the threshold. This proves that the ray-tracing approach is able to identify the damage location accordingly.

It should be noted that in Figure 15, corresponding to scenario D4, no paths pass through the location D4’. In such cases if the damage was present at D4’, it could not be localized with the ray-tracing approach based on edge reflections for the current AS configuration. For this purpose, optimization of the sensor and actuator placement is a key aspect which is identified as another area of future work.

**Figure 16:**
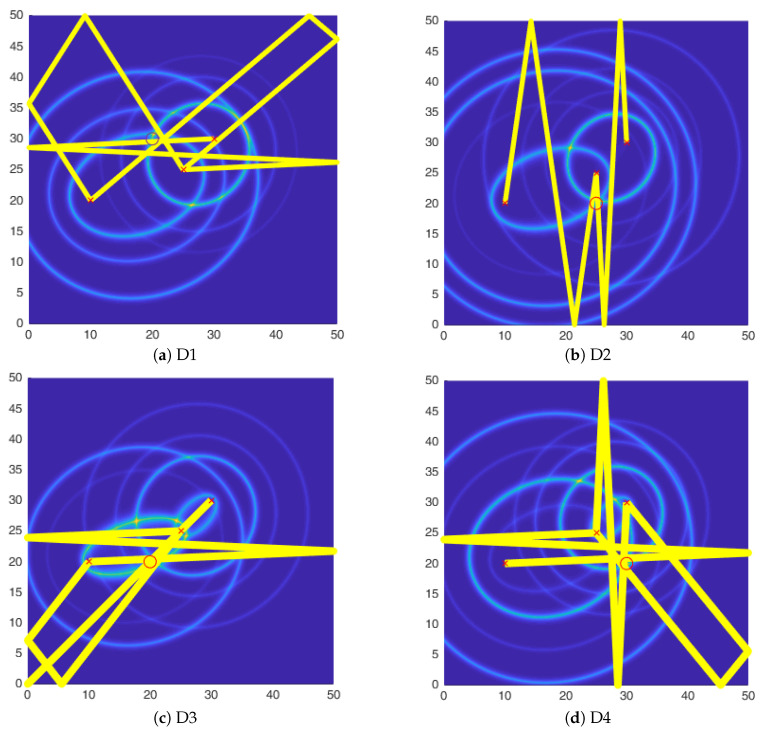
Damage localization using two-step methodology.

Figure 16 shows the final damage localization after the two-step method for the four damage scenarios. It should be noted that the damage map cannot be integrated into one DI value, as the values obtained from the two-steps are not in the same units. Therefore, the information from the two methods is fused qualitatively at the decision level in order to obtain the accurate damage localization.

## 5. Conclusions

GW-based damage detection is one of the most promising techniques for SHM for plate-like structure. The FBG based sensors in the edge-filtering approach have shown potential for sensing of the GW. Due to their desirable properties, the FBG sensors may indeed find many applications in GW based SHM. Unfortunately, the directionality of the FBG sensing and the passive nature of the FBG sensors makes it impossible for the existing methods to be applied to the data collected using these sensors. Therefore, this paper proposes a two-step methodology for FBG sensors for GW based damage detection which overcomes the shortcomings of the FBG. The paper presents a proof of concept of the methodology for an aluminum plate with simulated damage scenarios.

In the first step the damage hotspots are detected using the amplitude weighted elliptical approach. In the absence of 3 AS pairs, the method is incapable of localizing the damage, but can only identify non-unique hotspots. In order to further localize the damage uniquely, the ray tracing approach based on edge reflections is applied. The results indicate that indeed the two-step methodology allows us to localize the damage accurately.

Firstly, the two-step method overcomes the passive nature of the FBG sensors (paucity in the AS pairs). Second, by using the amplitude weighted elliptical approach, the directional sensitivity is incorporated in the algorithm. Furthermore, in the damage localization step, by taking the ratio of the RMS values between the healthy and damage scenarios, the effect of the directionality is overcome (both healthy and damage scenarios for the same path have same incident angle). These features of the methodology make it ideal for application for FBG based GW data.

It is acknowledged, that more in-depth study is needed before application of the methodology on real structures is possible. The aim of this paper is to provide a proof-of concept of the developed methodology. The identified areas of further research are to expand the study for different frequencies of GW excitation, investigate the optimal sensor placement for the sensors and actuator location, and investigate the suitability of the method for anisotropic materials and structures with complex geometry. Another area for future work is to investigate the suitability of the method for multiple damage scenarios and determine the sensitivity of the edge reflection method to small levels of damage (small mass) as well as determining the value of threshold for *A_i_* statistically.
